# Impact of SARS-CoV-2-Pandemic on Mental Disorders and Quality of Life in Patients With Pulmonary Arterial Hypertension

**DOI:** 10.3389/fpsyt.2021.668647

**Published:** 2021-06-24

**Authors:** Da Hee Park, Jan Fuge, Tanja Meltendorf, Kai G. Kahl, Manuel J. Richter, Henning Gall, Hossein A. Ghofrani, Jan C. Kamp, Marius M. Hoeper, Karen M. Olsson

**Affiliations:** ^1^Department of Respiratory Medicine, Hannover Medical School, Hannover, Germany; ^2^Biomedical Research in Endstage and Obstructive Lung Disease Hannover (BREATH), German Center for Lung Research (DZL), Hannover, Germany; ^3^Department of Psychiatry, Social Psychiatry, and Psychotherapy, Hannover Medical School, Hannover, Germany; ^4^Department of Internal Medicine, Justus Liebig University Giessen, Universities of Giessen and Marburg Lung Center, Giessen, Germany; ^5^Member of the German Center for Lung Research (DZL), Giessen, Germany

**Keywords:** Covid-19 pandemic, pulmonary arterial hypertension, mental disorder, depression, anxiety

## Abstract

**Background/Objective:** Covid-19 pandemic may affect mental health and quality of life (QoL) in patients with pulmonary arterial hypertension (PAH). We assessed changes in anxiety and depression, quality of life (QoL) and self-described impact of Covid-19 in patients with PAH during the Covid-19 pandemic.

**Methods:** This study included 152 patients with PAH from two German referral centers. Anxiety and depression were assessed using the Hospital Anxiety and Depression Scale (HADS-A and HADS-D) at two different timepoints before and during the Covid-19 pandemic with a median of 232 days between baseline and follow-up. QoL was assessed using EQ-5D and emPHasis-10. Perceived impact of Covid-19 and related regulations and measures were assessed using a set of specific questions and statements.

**Results:** More than two thirds of patients had an unsuspicious HADS-A and HADS-D. Median scores did not differ from baseline for both HADS-A and HADS-D (*p* = 0.202; *p* = 0.621). Overall, no significant changes in HADS-A or HADS-D categories from baseline to follow up were observed (*p* = 0.07; *p* = 0.13). QoL did not change between baseline and follow-up. The Covid-19 pandemic had little impact on access to medical care and established PAH therapy. Patients were in agreement with governmental measures and regulations and felt sufficiently safe.

**Conclusion:** First waves of Covid-19 pandemic had little impact on anxiety, depression and QoL in patient with PAH. Established PAH therapy and access to medical care were not affected. Further studies on the impact of prolonged duration of the ongoing Covid-19 pandemic are needed.

## Introduction

Since the first confirmed case of coronavirus disease 19 (Covid-19) caused by the novel beta-coronavirus SARS-CoV-2 was reported in January 2020, the disease has had great impact on every aspect of German society (1). In March 2020 the World Health Organization (WHO) declared Covid-19 a pandemic. The incidence in Germany has increased to over 2.29 Mio. cases including over 60,000 deaths so far (2). In response to the rising number of cases and deaths attributed to Covid-19, nationwide governmental measures such as mandatory quarantine, lockdown and physical distancing guidelines were implemented. Covid-19 has greatly affected the economy, employment and public health. Recent studies indicate implications of Covid-19 pandemic on mental health with higher levels of perceived stress, anxiety and depression (3–7). Bauerle et al. found increased anxiety and depression symptoms during the Covid-19 outbreak Germany. Covid-19 related fear proved to be a strong predictor of changes in mental health before and during the Covid-19 pandemic (8).

Pulmonary arterial hypertension (PAH) is a rare progressive pulmonary disease characterized by remodeling of pulmonary vasculature leading to increased pulmonary vascular resistance and left untreated to death by right heart failure (9, 10). Patients with PAH experience symptoms such as dyspnea on exertion, fatigue, syncope and clinical signs of heart failure impairing physical activities and quality of life (QoL) (11). Despite vast advances in therapeutic options over the last decades and subsequent improvements in clinical outcomes, PAH remains a life-threatening illness (12).

Only few studies have addressed the prevalence of psychiatric disorders in PAH patients (13, 14). Lowe et al. demonstrated a high prevalence of anxiety and depression with disease progression (15). Frequency of adjustment disorders, anxiety and depression correlate with intensity of symptoms and functional impairment in patients with PAH (16). Olsson demonstrated a higher prevalence of anxiety and depression disorders in PAH compared to levels in the German general public (17, 18). Yogeswaran et al. found significant negative impact of the Covid-19 pandemic on access to medical care for patients with suspected pulmonary hypertension (PH) due pandemic related restrictions in Germany (19).

The present study aimed to assess changes on mental health and QoL during the Covid-19 pandemic and the impact of the pandemic in a PAH cohort with a higher prevalence of anxiety and depression disorder.

## Methods

This prospective, exploratory observational multicenter study included patients with confirmed PAH of a cohort previously described in the PEPPAH-study (18), at two participating PH referral centers (Hannover Medical School and University of Giessen, both in Germany). This study was conducted from May 7th, 2020 to August 4th, 2020 by a self-administrated questionnaire. All patients gave written informed consent. The study was approved by local institutional review board (Nr. 8540_BO_K_2019). The questionnaire was mailed to study participants together with a free postage reply envelope.

### Patient Setting and Clinical Parameters

All patients who had previously participated in the PEPPAH study where reapproached *via* mail. Patients were selected based on diagnosis of PAH according to current criteria (9) and age ≥ 18 years. Baseline assessment defined as timepoint of PEPPAH also included hemodynamics from right heart catherization at time of diagnosis, 6-min walk distance (6MWD), WHO functional class (FC), and serum levels of N-terminal fragment of pro-brain natriuretic peptide (NT-proBNP). Follow up assessment was defined as last available clinical data not older than 90 days at time of questionnaire. Risk assessment for our cohort was based on three variables FC, 6MWD, and BNP/NT-proBNP as previously described (20, 21). Each variable was graded with a number as low risk (1), intermediate risk (2) and high risk (3). The average risk was calculated by dividing the sum of the grades by the number of available variables and rounding to the next integer (22). Hospital-wide Covid-19 regulations restricted in-person care to urgent or emergency outpatient visits. To compensate for this, we provided remote monitoring of symptoms, general condition and medication *via* phone or email for our outpatients.

### Assessment of Mental Disorders and Covid-19 Implications

We assessed symptoms of anxiety and depression, health-related quality of life (HRQoL) and Covid-19 pandemic related questions. Participants completed the following set of questionnaires: the Hospital Anxiety and Depression Scale (HADS) (23), the EuroQoL EQ-5D subscored in EQ-5D-3L and EQ-VAS (24), emPHasis-10 (25), and Covid-19 pandemic related questions (**Table 2**). The HADS questionnaire is divided into subscales for anxiety (HADS-A) and depression (HADS-D), each containing 7 questions for a maximum of 21 points per subscale. The higher score indicating a more severe anxiety or depression. Zigmond and Snaith (23) proposed a score >11 per subscale to be associated with significant anxiety or depression. A cut-off score of 8 or above for both HADS-A and HADS-D was validated, showing probable signs of anxiety or depression (26, 27). The EQ-5D-3L has five dimensions. Higher scores indicate a better quality of life. The EQ-VAS is a scale with a range from 0 (the worst health you can imagine) to 10 (the best health you can imagine). The emPHasis-10 is a PH specific HRQoL questionnaire. The emPHasis-10 score ranges from 0 to 50 with higher scores indicating worse HRQoL. Covid-19 related questions applying a 6 point Likert scale (1 = strongly disagree to 6 = strongly agree) ranged from subjective impact on everyday life, general anxiety to PH-therapy and opinion on governmental regulations during the Covid-19 pandemic (see Supplementary Material). Frequency of outpatient visits at the time of this study were compared to the same timeframe in 2019.

### Statistical Analysis

IBM SPSS Statistics (version 27.0, IBM Corp., Armonk, New York) and Stata 13.0 (State Corp LP, College Station, Texas, USA) statistical software programs were used for statistical analysis. All analyses were descriptive and there was no formal study hypothesis. Categorical data were presented as counts with percentages. Continuous variables were presented as median with the first and third quartile (Q1 and Q3) or as mean and standard deviation (SD). For group comparisons Chi-square, Fisher's exact-test or McNemar Bowkers-Test, paired *t*-test or Wilcoxon-test were used as appropriate. All tests were two-sided, a *p*-value of < 0.05 was considered statistically significant.

## Results

The questionnaires were employed on the May 7th, 2020 for Hannover and May 18th, 2020 for Giessen, respectively. The last questionnaire was returned on July 3rd, 2020. Data collection was conducted ~7 months after the PEPPAH study. A total of 204 patients were approached in this study. One hundred and fifty-two patients (75%) (27 from Giessen and 125 from Hannover) agreed to participate and returned their self-administered questionnaires. One patient died shortly before mailing arrived and one patient refused participation. Patient characteristics are shown in Table 1. The majority of patients were female (*n* = 111, 73%); median age was 58 (49–67) years. All patients were treated with PAH medications, most of them (74%) with dual combination therapy.

**Table 1:** Patient characteristics.

All patients—*n*	152
Site—*n* (%)	
- Hannover	125 (82%)
- Giessen	27 (18%)
Sex—*n* (%)	
- Female	111 (73%)
- Male	41 (27%)
Age—median (IQR)	58 (49–67)
Diagnosis—*n* (%)	
−1.1 IPAH	80 (53%)
−1.2 HPAH	17 (11%)
−1.4 Associated PAH	53 (35%)
−1.6 PVOD/PCH	2 (1%)
PH-Therapy—*n* (%)	
- PDE5i	126 (83%)
- ERA	121 (80%)
- PCA (Selexipag and Treprostinil)	42 (28%)
- TKI	5 (3%)
- sGCs	15 (10%)
- CCB	21 (14%)
Time—median (IQR)	
- From diagnosis to baseline—years	8 (4–14)
- From baseline to follow up—days	232 (204–240)

*Continuous variables are stated as median and interquartiles ranges (IQR) and categorical variables are stated as n and percent (%), unless stated otherwise. I/HPAH, idiopathic or heritable pulmonary arterial hypertension; PVOD/PCH, pulmonary veno-occlusive disease/pulmonary capillary hemangiomatosis; PDE5i, phosphodiesterase-5 inhibitors; ERA, endothelin receptor antagonists; PCA, prostacyclin receptor agonists; TKI, tyrosine kinase inhibitor; sGCs, stimulators of soluble guanylate cyclase; CCB, calcium channel blocker*.

### Clinical Parameters

ESC/ERS risk status, disease severity (FC, 6MWD, NTproBNP), reported subjective condition and stair-climbing performance did not differ between baseline and follow-up (see Table 2).

**Table 2:** Comparison of psychological, clinical parameters and HRQoL.

	**Baseline**	**Follow-up**	***p*-value**
HADS-A-Score—median (IQR)	6 (2–9)	6 (3–8)	0.202
- Unsuspicious	100 (66%)	103 (68%)	0.070^*^
- Suspected	26 (17%)	33 (22%)	
- Probable	26 (17%)	16 (11%)	
HADS-D-Score—median (IQR)	5 (2–7)	4 (2–7)	0.621
- Unsuspicious	117 (77%)	118 (78%)	0.135^*^
- Suspected	15 (10%)	22 (15%)	
- Probable	20 (13%)	12 (8%)	
emPHasis10—median (IQR)	14 (8–25)	18 (11–29)	0.009
EQ5D-3L—median (IQR)	8 (7–9)	8 (6–9)	0.181
EQ-VAS—median (IQR)	7 (6–8)	7 (5–8)	0.366
Subjective condition—*n* (%)			0.899^*^
- Better	5 (9%)	15 (10%)	
- Stable	44 (79%)	116 (76%)	
- Worsened	7 (13%)	21 (14%)	
Stair climbing—*n* (%)			0.276^*^
- >2	11 (33%)	35 (23%)	
−2	8 (24%)	52 (35%)	
−1	13 (39%)	47 (31%)	
−0	1 (3%)	16 (11%)	
WHO FC—*n* (%)			0.766^*^
- I	16 (11%)	6 (12%)	
- II	72 (48%)	26 (51%)	
- III	63 (41%)	19 (37%)	
6MWD—median (IQR)	452 (365–532)	457 (367–528)	0.132
NT-proBNP (ng/l) —median (IQR)	179 (84–419)	314 (123–1024)	0.836
ESC/ERS risk status—*n* (%)			-
- Low	40 (67%)	41 (55%)	
- Intermediate	20 (33%)	33 (44%)	
- High	0 (0%)	1 (1%)	

*Continuous variables are stated as median and interquartiles ranges (IQR) and categorical variables are stated as n and percent (%), unless stated otherwise*.

* *Chi^2^-Test HADS, Hospital Anxiety and Depression Scale; WHO FC, World Health Organization Functional Class; 6MWD, six-minute walking distance; NT-proBNP, N-terminal fragment of pro-brain natriuretic peptide; ESC, European Society of Cardiology, ERS, European Respiratory Society*.

### Psychological Effects During the Covid-19 Pandemic

**Figure 1:**
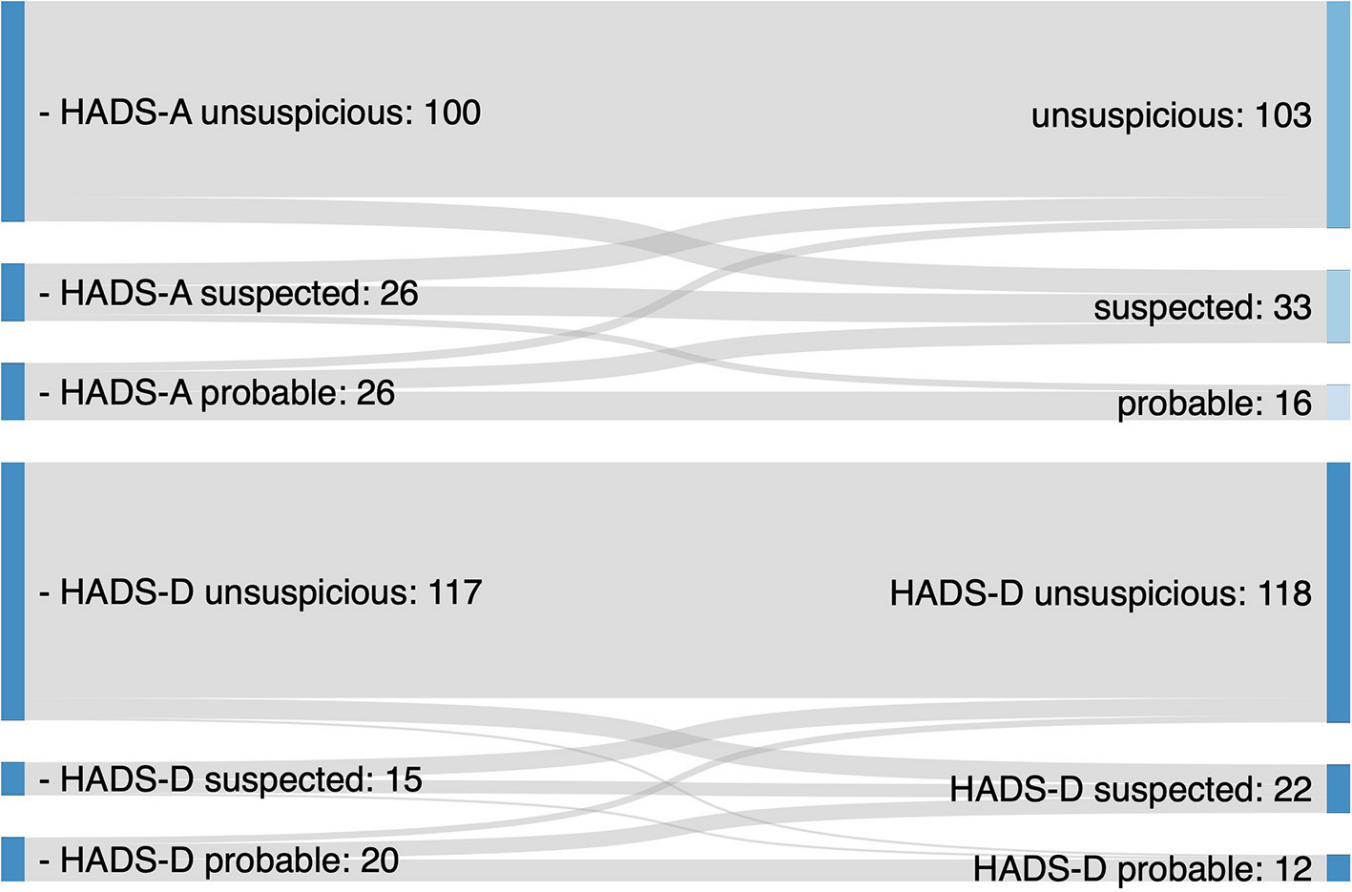
Changes in HADS-A and HADS-D categories prior to and during the Covid-19 pandemic.

The majority of patients (68%) had an unsuspicious HADS-A with an overall median score of 6. One third were classified suspected (22%) or probable (11%) for an anxiety disorder. Median HADS-A score did not differ from baseline (*p* = 0.202). For HADS-D the majority of patients was unsuspicious (78%), and 22% had signs of suspected or probable depression disorder (see Table 2). Median HADS-D score did not differ from baseline (*p* = 0.621). Overall, no significant changes in HADS-A or HADS-D categories from baseline to follow up were observed (*p* = 0.07 and *p* = 0.13, respectively). For both HADS-A and HADS-D, more patients were classified with a suspected score at follow-up and less people with a probable score. Transitions in HADS-A and HADS-D categories are shown in Figure 1. For HADS-A, 25 patients (16%) and HADS-D 21 patients (14%) changed categories. Measured by emPHasis-10, HRQoL was significantly lower on follow-up (18 points) than at baseline (14 points). HRQoL measured by EQ5D-3L and EQ-VAS did not differ between baseline and follow-up (see Table 2).

### Covid-19 Pandemic Related Questionnaire and Outpatient Care

**Figure 2:**
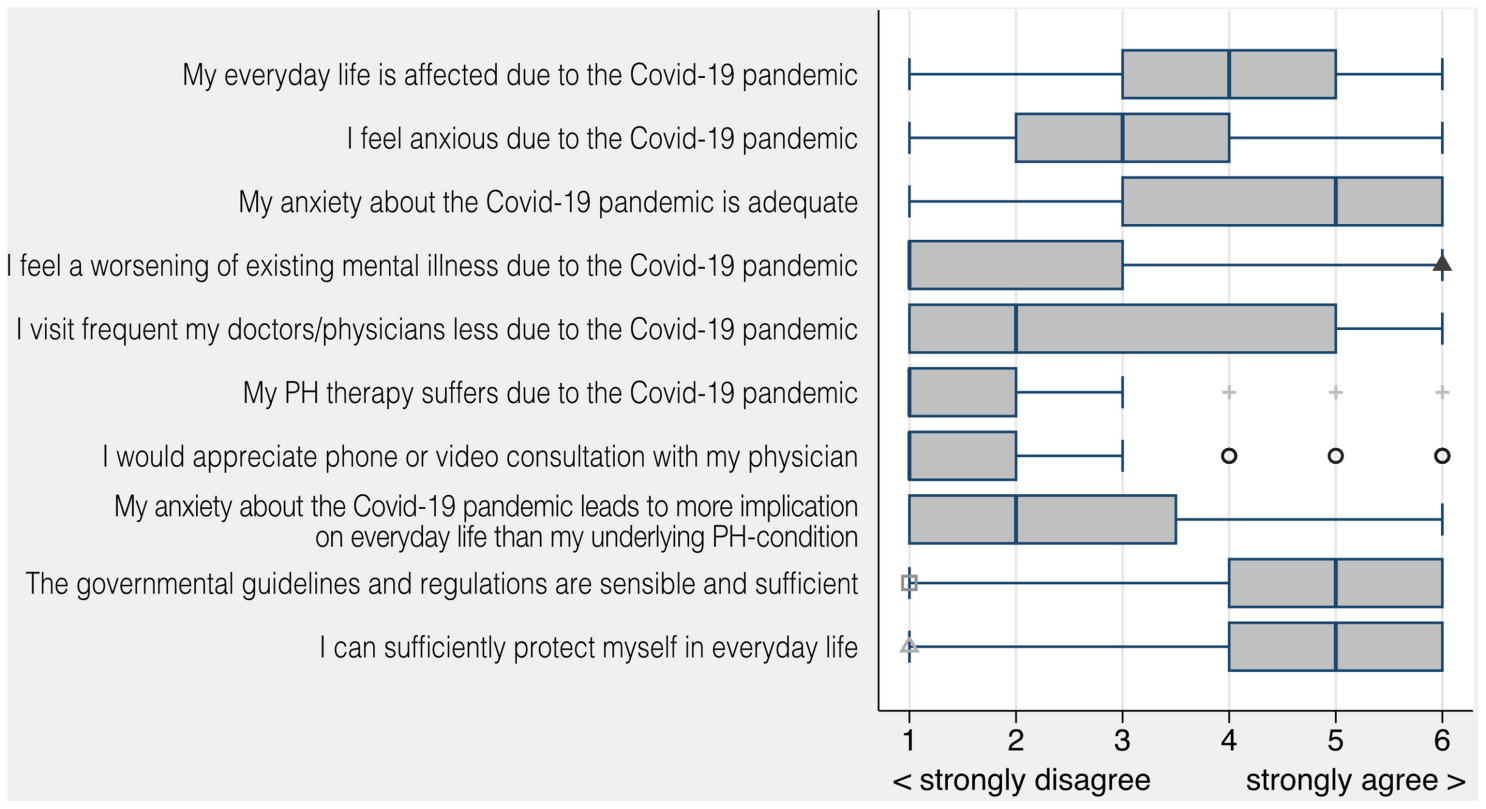
Covid-19 pandemic specific questions; Likert scale 1 = strongly disagree to 6 = strongly agree.

The majority of patients felt affected in their everyday life (mean 3.93 ± 1.55) due to the pandemic. Anxiety about Covid-19 did not have implications on patient's everyday life (2.44 ± 1.65). The majority of patients stated that their PH therapy did not suffer during the Covid-19 pandemic (mean 1.70 ± 1.27). Most of them were in agreement with governmental regulations and the ability to self-protect during the Covid-19 pandemic (mean 4.89 ± 1.31; mean 4.76 ± 1.40, see Figure 2). The Covid-19 pandemic showed little impact on fear of worsening of existing mental illness (mean 1.89 ± 1.23). Patients did not see a need for more phone or video consultations from their physician (mean 1.89 ± 1.51). Access to medical care and medication was perceived as unproblematic (Likert scales prior and during the Covid-19 pandemic for medical care 1.46 vs. 2.16 and medication 1.53 vs. 1.79). Outpatient visits during Covid-19 pandemic decreased by 46% from March to June 2020 reaching its lowest rate in March 2020 with 32% of ambulatory visits compared to the same timeframe in 2019.

## Discussion

The present study showed a limited impact of the Covid-19 pandemic on anxiety, depression and QoL in patients with PAH. Olsson identified higher prevalence of depression and panic disorder in patients with PAH contributing to impaired QoL (18). Psychological distress in PAH results from impact on daily life due to physical limitations, unclear prognosis and unemployment (28, 29). Our study revealed transitions between HADS-A and HADS-D categories from baseline to follow-up. Whereas there were no significant changes between groups overall, individual patients moved from unsuspicious to suspected and for HADS-D even one patient from unsuspicious to probable, indicating an individual risk of mental health deterioration. For HADS-A these individual transitions also showed a trend for overall changes between category groups (*p* = 0.07). It remains unclear if changes in mental health assessment stem from normal range variations or if they imply an impact of the Covid-19 pandemic.

Overall QoL did not differ prior to and during the Covid-19 pandemic, which may have been related—at least partly—to the fact that access to medical care was not impaired. While some changes in QoL were detected using emPHasis-10, QoL remained stable with a median well below 20 points for both baseline and follow up. The changes were mainly driven by emPHasis-10 subscores for confidence in public places (subscore 7) and independency (subscore 9), indicating effects of Covid-19 pandemic related regulations such as social distancing measures.

In a recent study Ammar et al. demonstrated negative effects on mental well-being and emotional status caused by social distancing and self-isolation during the Covid-19 pandemic using an international cross-disciplinary online survey. However, only 21% of the participants came from Europe and there was (and is) a wide regional variation in Covid measures and access to medical care (30). In line with our study, unpublished data from a lung cancer cohort of our hospital showed little effect on mental health during the Covid-19 pandemic which might also be due to the consistent and stable medical care of this patient population.

Attitude toward governmental measures might have an impact on mental health during the Covid-19 pandemic. On March 22nd, 2020, lockdown measures such as restrictions on gatherings, social distancing restrictions and closure of non-essential businesses were put in place in Germany. Our study showed that our patients were mostly in agreement with these governmental regulations. While patients felt an impact on their everyday life, they were not afraid of worsening of mental illness. While Yogeswaran et al. (19) saw impacts on initial PH care and treatment initiation in patients with newly diagnosed PH, our cohort reported little impact on established PH-therapy. Even though we saw reduction in outpatient visits during the time of the study we can presume that established remote patient care was deemed sufficient in patients with stable disease.

Popularity of telemedicine has risen during the Covid-19 pandemic. Kayser et al. demonstrated video consultations as a valuable tool in quality health care in a selected patient group during the Covid-19 pandemic (31). Phone and email consultations as well as a repeat prescription online ordering service developed into a mainstay of patient care at our center during the Covid-19 pandemic and were readily accepted by our patients. This might explain why we did not see a need for additional video consultations.

There are several limitations to this study. The assessment was limited to the first wave of infection in Germany in a time of decreasing infections and a falling infection curve. This and the relatively short observation period may have led to underestimation of the psychological impact on the pandemic on patients with PAH. Still, the Covid-19 pandemic continued after a summer with low infection rates with a second wave and increasing rates in autumn 2020. Impact of the continuing Covid-19 pandemic including aspects of prolonged duration, further isolation and restrictions, economical and personal burden of family members or friends are not represented in this study. Availability of clinical parameters were limited. Questionnaires for both time points were sent by mail and interviews were conducted *via* phone. Ambulatory visits were not obligatory so nearby clinical data to the time points of the study were not always available. In addition, data on pre-existing mental disorders were not available.

The psychiatric tools used in the present study have not been validated in patients with PAH. HADS is a widely used tool to assess anxiety and depression in non-psychiatric patients. In patients with heart disease, HADS shows high specificity for the detection of major depression but a limited sensitivity for mild depression disorder (32). Another limitation is potential selection bias. Although 75% of our patient responded, we cannot be certain that the results would have been different with a higher response rate. Further bias might be due to stigma or self-stigma which may cause serious problems in patients with mental disorders. We cannot rule out the possibility that patients with PAH are underscored in the administered self-rating scales, particularly the HADS (33–35).

In conclusion, the Covid-19 pandemic had little impact on intensity of anxiety or depression and overall QoL in our cohort of patients with PAH. While the number outpatient visits declined, access to medical care and established PAH therapy was felt appropriate. The impact of the ongoing Covid-19 pandemic with prolonged lockdown measures remains unknown and deserves further investigation.

## Data Availability Statement

The raw data supporting the conclusions of this article will be made available by the authors, without undue reservation.

## Ethics Statement

The studies involving human participants were reviewed and approved by MHH Ethikkommission. The patients/participants provided their written informed consent to participate in this study.

## Author Contributions

DHP and JF were responsible for study design, implementation of the study, data collection, statistical analysis, data interpretation, and drafting the manuscript. KO and MH were responsible for study design, implementation of the study, data interpretation, and critically revising the manuscript. JK, TM, and KK were responsible for data interpretation and revising the manuscript. HAG, HG, and MR were responsible for implementation of the study, data collection, data interpretation, and revising the manuscript. All authors contributed to the article and approved the submitted version.

## Conflict of Interest

KK has received honoraria for consultations and/or lectures from Eli Lilly, Janssen, Lundbeck, Neuraxpharm, Otsuka, Pfizer, Servier, Schwabe, Takeda, and Trommsdorff/Ferrer. HG has received personal fees from Actelion, AstraZeneca, Bayer, BMS, GSK, Janssen-Cilag, Lilly, MSD, Novartis, OMT, Pfizer, United Therapeutics, outside the submitted work. HAG has received fees from Actelion, Bayer, Gilead, GSK, MSD, Pfizer and United Therapeutics, outside the present work. MH has received fees for lectures and/or consultations from Acceleron, Actelion, Bayer, GSK, Janssen, MSD and Pfizer, all outside the present work. KO has received fees for lectures and/or consultations from Acceleron, Actelion, Bayer, Janssen, MSD, United Therapeutics, GSK and Pfizer, all outside the present work. The remaining authors declare that the research was conducted in the absence of any commercial or financial relationships that could be construed as a potential conflict of interest.

## Abbreviations

6MWD: 6-minute walk distance
BNP: brain natriuretic peptide
Covid-19: coronavirus disease 19
FC: World Health Organization functional class
HADS: Hospital Anxiety and Depression Scale
HRQoL: health-related quality of life
NT-proBNP: N-terminal fragment of probrain natriuretic peptide
PAH: Pulmonary arterial hypertension
PEPPAH: Psychische Einschränkungen bei Patienten mit pulmonal arterieller Hypertonie (Prevalence of mental disorders and impact on quality of life in patients with pulmonary arteriaI hypertension)
PH: pulmonary hypertension
QoL: quality of life
SARS: Severe acute respiratory syndrome
SD: standard deviation
WHO: World Health Organization.
