# Supplementary material for: Impact of SARS-CoV-2-Pandemic on Mental Disorders and Quality of Life in Patients With Pulmonary Arterial Hypertension
Source: Front Psychiatry. 2021 Jun 24;12:668647. doi: 10.3389/fpsyt.2021.668647 (PMC8263927; doi:10.3389/fpsyt.2021.668647)
Supplement: Supplementary file 1 [file Data_Sheet_1.pdf]

Supplement

Specific questions regarding the Sars-CoV-2 Pandemic (Coronavirus)

	Strongly disagree 1	2	3	4	5	Strongly agree 6
My everyday life is affected due to the Covid-19 pandemic	○	○	○	○	○	○
I feel anxious due to the Covid-19 pandemic	○	○	○	○	○	○
My anxiety about the Covid-19 pandemic is adequate	○	○	○	○	○	○
I feel a worsening of existing mental illness due to the Covid-19 pandemic	○	○	○	○	○	○
I visit my doctors/physicians less frequent due to the Covid-19 pandemic	○	○	○	○	○	○
My PH-therapy suffers due to the Covid-19 pandemic	○	○	○	○	○	○
I would appreciate phone or video consultations with my physician	○	○	○	○	○	○
My anxiety about the Covid-19 pandemic leads to more implications on everyday life than my underlying PH-condition	○	○	○	○	○	○
The governmental guidelines and regulations are sensible and sufficient	○	○	○	○	○	○
I can sufficiently protect myself in everyday life	○	○	○	○	○	○

The following two questions relate to the time **PRIOR** to the Covid-19 pandemic

	Very easy 1	2	3	4	5	Very difficult 6
How easy was it for you to access medical help prior to the Covid-19 pandemic?	○	○	○	○	○	○
How easy was it for you to obtain your medications prior to the Covid-19 pandemic?	○	○	○	○	○	○

The following two questions relate to the time now, **DURING** the Covid-19 pandemic

	Very easy 1	2	3	4	5	Very difficult 6
How easy was it for you to access medical help during the Covid-19 pandemic?	○	○	○	○	○	○
How easy was it for you to obtain your medications during the Covid-19 pandemic?	○	○	○	○	○	○

Original German Version: Spezifische Fragen zur Sars-CoV-2-Pandemie (Coronavirus)

	Trifft gar nicht zu 1	2	3	4	5	Trifft voll zu 6
Ich fühle mich durch die Corona-Pandemie beeinträchtigt	O	O	O	O	O	O
Ich empfinde aufgrund der Corona-Pandemie Angst	O	O	O	O	O	O
Ich empfinde meine Angst vor der Corona-Pandemie als angemessen	O	O	O	O	O	O
Ich bemerke eine Verschlechterung einer bestehenden psychischen Erkrankung durch die Corona-Pandemie	O	O	O	O	O	O
Ich gehe durch die Corona-Pandemie weniger oft zum Arzt	O	O	O	O	O	O
Meine PH-Therapie leidet unter Corona-Pandemie	O	O	O	O	O	O
Ich wünsche mir eine Telefon- oder Videosprechstunde mit meinem Arzt	O	O	O	O	O	O
Meine Angst vor Corona führt zu größeren Einschränkungen in meinem Alltag als meine pulmonale Hypertonie	O	O	O	O	O	O
Die empfohlenen Vorsichtsmaßnahmen der Regierung empfinde ich als sinnvoll und ausreichend	O	O	O	O	O	O
Ich kann mich in meinem Alltag ausreichend schützen	O	O	O	O	O	O

Die folgenden zwei Fragen beziehen sich auf die Zeit **VOR** der Corona-Pandemie

	Sehr leicht 1	2	3	4	5	Sehr schwer 6
Wie leicht war es für sie, medizinische Hilfe zu bekommen?	O	O	O	O	O	O
Wie leicht war es für sie, Medikamente für ihre PH zu bekommen?	O	O	O	O	O	O

Die folgenden zwei Fragen beziehen sich auf die Zeit jetzt, **WÄHREND** der Corona-Pandemie

	Sehr leicht 1	2	3	4	5	Sehr schwer 6
Wie leicht ist es für sie, medizinische Hilfe zu bekommen?	O	O	O	O	O	O
Wie leicht ist es für sie, Medikamente für ihre PH zu bekommen?	O	O	O	O	O	O
